# Spatial modelling and mapping of female genital mutilation in Kenya

**DOI:** 10.1186/1471-2458-14-276

**Published:** 2014-03-25

**Authors:** Thomas NO Achia

**Affiliations:** 1 School of Public Health, University of Witwatersrand, Johannesburg, South Africa; 2 School of Public Health, University of the Western Cape, Western Cape, South Africa

**Keywords:** Spatial hierarchical Bayesian analysis, Female genital mutilation, Integrated Nested Laplace Approximation, Disease mapping

## Abstract

**Background:**

Female genital mutilation/cutting (FGM/C) is still prevalent in several communities in Kenya and other areas in Africa, as well as being practiced by some migrants from African countries living in other parts of the world. This study aimed at detecting clustering of FGM/C in Kenya, and identifying those areas within the country where women still intend to continue the practice. A broader goal of the study was to identify geographical areas where the practice continues unabated and where broad intervention strategies need to be introduced.

**Methods:**

The prevalence of FGM/C was investigated using the 2008 Kenya Demographic and Health Survey (KDHS) data. The 2008 KDHS used a multistage stratified random sampling plan to select women of reproductive age (15–49 years) and asked questions concerning their FGM/C status and their support for the continuation of FGM/C. A spatial scan statistical analysis was carried out using SaTScan™ to test for statistically significant clustering of the practice of FGM/C in the country. The risk of FGM/C was also modelled and mapped using a hierarchical spatial model under the Integrated Nested Laplace approximation approach using the INLA library in R.

**Results:**

The prevalence of FGM/C stood at 28.2% and an estimated 10.3% of the women interviewed indicated that they supported the continuation of FGM. On the basis of the Deviance Information Criterion (DIC), hierarchical spatial models with spatially structured random effects were found to best fit the data for both response variables considered. Age, region, rural–urban classification, education, marital status, religion, socioeconomic status and media exposure were found to be significantly associated with FGM/C. The current FGM/C status of a woman was also a significant predictor of support for the continuation of FGM/C. Spatial scan statistics confirm FGM clusters in the North-Eastern and South-Western regions of Kenya (p < 0.001).

**Conclusion:**

This suggests that the fight against FGM/C in Kenya is not yet over. There are still deep cultural and religious beliefs to be addressed in a bid to eradicate the practice. Interventions by government and other stakeholders must address these challenges and target the identified clusters.

## Background

Female Genital Mutilation/Cutting (FGM/C) has been described as the partial or total removal of the female genitalia or other injury to the female genital organs for cultural or other non-therapeutic reasons [1-3]. The practice is prevalent across the world with an estimated 100–140 million girls and women forcibly circumcised [2,4]. A greater number of women have also been socialized to embrace FGM/C as an integral part of womanhood. Many girls and women bleed to death, or suffer disabilities that make it difficult for them to give birth normally, resulting in the death of the baby or health complications for those mothers and babies that survive.

The complications faced and challenges presented in giving appropriate care to circumcised women is widely reported in the literature. In the short term such complications include shock, haemorrhage, severe pain, infection, urinary retention and psychological sequalae [5-9]. Long term complications include dermoid cysts at the site of the amputated clitoris, urinary problems such as pain at micturition, dribbling urine incontinence and poor urinary flow, an increased risk of childbirth complications and new-born deaths [3,4,10]. Other long term complications include fibrosis, primary infertility, disorders of desire/libido, arousal, pain/discomfort, and inhibited orgasm [9,11-13].

It is against this background that the Committee on the Elimination of All Forms of Discrimination against Women issued its General Recommendation on Female Circumcision (General Recommendation No 14) that calls upon states to take appropriate and effective measures with a view to eradicating the practice and requests them to provide information about measures being taken to eliminate FGM/C in their reports to the Committee [14,15].

Kenya is home to an estimated 38.3 million people from more than 30 ethnic groups. It is also estimated that FGM/C is practiced in more than three quarters of the country, with prevalence of the practice varying widely from one ethnic group to another. Prevalence rates differ by provinces with rates of 26.5%, 33.8% and 32.1% recorded in Central, Nyanza and Rift Valley provinces respectively. Recent data suggest a decline from highs of 38% in 1998, to 32% and 27% in 2003 and 2008–9 respectively. There are also marked age variations in FGM/C with 15% of women aged 15–19 years and 49% of those aged 45–49 years circumcised [16,17]. Currents statistics also show that rural women are more likely than urban women to have been circumcised.

No articles were found in the literature survey that assessed regional, or even localized clusters of FGM/C, or the intention to continue the practice of FGM/C in Kenya. Only a limited number of studies globally have utilized spatial analytics in studying FGM/C [18-20]. The primary aim of this study was to map the geographical variations in the practice of FGM/C in Kenya, and the existing support for the continuation of the practice within the country. This study was also carried out with the aim of detecting clustering of the practice and to determine whether the distribution of the practice reflected significant clustering or chance variability in the practice. A broader goal of the study was to identify ‘hotspots’ to base future research on to better understand determinants of FGM/C practice.

## Methods

### The data

The data used in this study was from the 2008 Kenya Demographic and Health Survey (KDHS). This was a national survey conducted by the National Council for Population and Development (NCPD) in collaboration with the Central Bureau of Statistics (CBS) and Macro International. The survey was national in scope and selected respondents using a two-stage stratified random sampling design and relied on a sampling frame maintained by the CBS. A questionnaire based on a model developed by the MEASURE DHS programme, with slight adjustments to reflect relevant issues in Kenya, was used to collect the survey data. Fieldwork was conducted between April and September 2008 and achieved an overall response rate of 97% of households and 96% of women aged 15–49 who were eligible for an individual interview.

The 2008 KDHS covered 8,444 women aged 15–49, and 3578 men aged 15–54 from 400 enumeration areas throughout Kenya. The survey collected detailed demographic and women’s health care information. The Geographical Positioning System (GPS) coordinates for Enumeration Areas (EAs) in both urban and rural areas were also collected.

### Ethical considerations

This study was based on secondary data with all participant identifiers removed. Survey procedures and instruments were approved by the Scientific and Ethical Review Committee of the Kenya Medical Research Institute (KEMRI) and by the Ethics Committee of the Opinion Research Corporation, Macro International Incorporated (ORC Macro Inc.), Calverton, USA. Ethical permission for use of the data in the present study was obtained from ORC Macro Inc.

Details concerning the data collection protocols are available on the Measures Demographic and Health Surveys (DHS) website (http://dhsprogram.com/).

### The response variables

The study considered responses to the questions “*Have you undergone FGM/C?*” and “*Should FGM/C be continued?*” as the two response variables of interest.

All the analysis in this paper was conducted for each of these variables separately.

### Covariates

Based on a survey of literature [3,21-32] and limitations inherent in the dataset used, we assessed the nature of the response variables and the following covariates: *woman’s age* (15–19, 20–24, 25–29, 30–34, 35–39, 40–44, 45–49)*; region of residence* (Nairobi, Central, Coast, Eastern, Nyanza, Rift Valley, Western, North Eastern)*; type of place of residence* (urban, rural)*; woman’s level of education* (no formal, primary, secondary, higher)*; religion* (Roman Catholic, Protestant/other Christian, Muslim, no religion, other)*; socioeconomic status* (poorest, poorer, middle, richer, richest)*; marital status* (never married, married/living together, separated)*; occupation* (not working, management, other)*; media exposure* (low, medium, high). The *current FGM/C status* of the woman was used as a predictor of support for the continuation of FGM/C.

A **media exposure index** was derived using a Principal Components (factor) Analysis (PCA) ) [33] and was based on responses to questions asked on *the frequency of watching television, the frequency of listening to radio,* and *the frequency of reading newspapers.* The respondents were then classified as having low, medium or high media exposure.

The lowest category of each ordinal covariate was used as the reference category in the Hierarchical modelling phase of the data analysis. Nairobi province, the seat of the capital city of Kenya, was used as the reference category for the covariate region of residence.

### Bivariate data analysis

In order to ensure that estimates derived in this study are representative at the national level, survey weights that were provided as part of the KDHS data set were accounted for in the statistical analysis. Basic frequencies and cross tabulations, correcting for weighting and stratification of the random samples, were carried out using the Stata SVY (survey) commands [34] for each response-covariate relationship. Design weighted F and Chi-square values were used to assess the nature of the association between the response variables and the covariates.

### Hierarchical spatial modelling

To model the relationship between the response variables and the predictors of interest, a hierarchical spatial modelling approach was used [35-38]. Hierarchical models allow us to borrow strength from neighbouring regions and the entire geographical region in order to stabilize estimates based on small, local sample sizes within sectors. Methodological details are described in detail elsewhere and are briefly outlined in the Appendix.

In this study, **Model 1** shall denote the (Bayesian) ordinary logistic regression model, **Model 2** the generalized linear mixed model with spatially unstructured random effects, **Model 3** the generalized linear mixed model with spatially structured random effects, and finally, **Model 4** the generalized linear mixed model with both the spatially structured and unstructured random effects. Each of these models was fitted to the dataset. However, we present unadjusted odds ratios for each of the covariates, adjusted results for the full model, with all covariates included and the best fitting model identified.

Bayesian inference was carried out using the *R* library *INLA*[39] which implements the Integrated Nested Laplace approximation approach for latent Gaussian models [40,41].

Model comparison and selection was carried out on the basis of the deviance information criterion (*DIC*), which is a measure of model complexity and fit. The DIC was used to compare complex hierarchical models [42]. Smaller values of *DIC* indicate a better trade-off between complexity and fit of the model.

### Spatial cluster detection

To identify significant FGM clusters we merged relevant household data, while adjusting for sampling weights, to obtain aggregated county level indicators of the proportion of women undergoing circumcision, and the proportion intending to have their eldest daughter circumcised. Spatial scan statistical analysis was carried out using SaTScan™ to test for statistically significant clustering of the practice of FGM in the country [43]. This program tests for spatial clustering using area (case and population at-risk) data, and outputs the location, approximate size and significance of identified clusters.

A data file containing raw FGM case and controls for the centroid (longitude and latitude coordinate) of each of the counties was obtained using GeoDa [44]. This data file was imported into SaTScan assuming a Bernoulli probability disease model (case, control and at-risk population data).

The presence of high-risk clusters was assessed for each of the categories of FGM practice. The *p-*values for maximum likelihood ratios were based on 9,999 Monte Carlo randomizations. An alpha level of 0.05 was used to assess statistical significance. Likelihood-ratio based test statistics and reported *p*-values account for multiple testing.

## Results

### Summary statistics

Table 1 presents the results of design weighted bivariate cross-tabulation of FGM/C with various covariates entertained. The national FGM/C prevalence rate stood at 28.2% (95% CI: 24.4-32.3%). We found significant bivariate associations between FGM/C and all the covariates considered (p < 0.001). The prevalence rate of FGM/C varied linearly from a high of 50.3% (95% CI: 43.3-57.3%) among women aged 45–49 years to 15.7% (95% CI: 11.8-20.6%) among women aged 15–19 years. We also found significant regional variation in FGM/C. The North Eastern province that borders Somalia and Ethiopia had an FGM/C prevalence rate of 97.6% (95% CI: 91.6-99.4%), more than triple the national prevalence rate. Other regions with high FGM/C prevalence rates were Nyanza 36.4% (95% CI: 23.0-52.2%), Eastern 36.4% (95% CI: 28.0-45.6%) and Rift Valley with 32.9% (95% CI: 25.7-41.0%). The results suggested a linear decline in FGM/C prevalence with education, socioeconomic status and media exposure. We also found a variation in FGM/C prevalence by religious affiliation. The prevalence of FGM/C among Muslims, women professing no religious affiliation and Roman Catholic women were all significantly higher than the national prevalence rates.

**Table 1:** Distribution of female genital mutilation by selected covariates

	**Women circumcised**		**FGM/C should continue**	
	**N(%)**		**N(%)**	
***Circumcised***				
No			5175(2.4)	
Yes			2434(30)	
***Age***		p < 0.001		p = 0.908
15-19	1633(15.7)		1543(9.9)	
20-24	1667(21.9)		1586(10.7)	
25-29	1368(26.1)		1300(10.7)	
30-34	1129(31)		1071(9)	
35-39	890(36.3)		842(10.7)	
40-44	704(40.8)		659(10.4)	
45-49	647(50.3)		608(10.9)	
***Region***		p < 0.001		p < 0.001
Nairobi	925(14.1)		884(6)	
Central	962(26.8)		947(5.3)	
Coast	978(11.8)		895(5)	
Eastern	1114(36.4)		1094(9.3)	
Nyanza	1226(36.4)		1059(20.1)	
Rift Valley	1241(32.9)		1188(6.8)	
Western	985(0.8)		953(1.7)	
North Eastern	607(97.6)		589(92.5)	
***Type of place of residence***		p < 0.001		p < 0.001
**Urban**	2529(4.4)		1153(22.1)	
**Rural**	5509(23.8)		3135(77.8)	
***Education***		p < 0.001		p < 0.001
No formal	1120(61.3)		1053(41.6)	
Primary	4172(28.9)		3905(8.2)	
Secondary	2043(21.4)		1969(7.8)	
Higher	703(12)		682(2.2)	
***Marital status***		p < 0.001		p = 0.002
Never married	2405(15.3)		2304(7.5)	
Married, living together	4817(34.4)		4546(11.7)	
Separated	816(31.1)		759(10.6)	
***Religion***		p < 0.001		p < 0.001
Roman Catholic	1624(30.4)		1549(8.7)	
Protestant/other Christian	4932(24.2)		4678(6.9)	
Muslim	1292(54.8)		1207(43.3)	
No religion	138(47.3)		128(30.2)	
Other	44(6.9)		39(5.1)	
***Socioeconomic status***		p < 0.001		p < 0.001
Poorest	1529(44.6)		1445(20.8)	
Poorer	1215(32.2)		1140(10.3)	
Middle	1407(30.2)		1334(10)	
Richer	1560(26.7)		1481(7.1)	
Richest	2327(15.7)		2209(7)	
***Media exposure***		p < 0.001		p < 0.001
Low	2299(44.3)		2161(19.7)	
Mid	2982(26.5)		2802(8.6)	
High	2743(18)		2632(5.3)	
***Occupation***		p < 0.001		p = 0.02
Not working	3535(24.4)		3327(11.9)	
Management	1446(21.1)		1369(6.4)	
Other	3038(35.1)		2895(10.4)	
**Total**	**8030(28.2)**		**7609(10.3)**	

Table 1 also presents the results of bivariate cross-tabulation of the support for continued FGM/C practice and the covariates considered. The proportion of women supporting the continuation of FGM/C at the time of the survey was surprisingly high and stood at 10.3% (95% CI: 8.4-12.4%). We also found significant bivariate associations between most of the covariates considered and support for the continuation of FGM/C. There was no association between the woman’s age and her support for the continuation of the practice. The proportion of circumcised women (30.0%, 95% CI: 25.4-35.0%), supporting the continuation of the practice was considerably higher than that of uncircumcised women (2.4%, 95% CI: 25.4-35.0%).

Support for the continuation of the practice was highest in the North Eastern (92.5%, 95% CI: 86.7-95.9%) and Nyanza (20.1%, 95% CI: 14.1-27.7%) provinces, where the practice is currently most prevalent.

### Spatial modelling and mapping

In Table 2 we present the results of fitting the hierarchical models to the FGM/C data. Similar results for responses to the question “*Should FGM/C continue*” are presented in Table 3. Tables 2 and 3 also present the effective number of parameters, *pD*, and the Deviance Information Criterion (*DIC*) for each of models entertained. Based on the *DIC* values, **Model 3**, the hierarchical model with spatially structured random effects was considered to be the best fitting model in each case.

**Table 2:** Odds ratios, adjusted odds ratios and 95% Credible Intervals (CI) for the association between FGM/C and the significant predictors

**Variable**		**Model 1**	**Model 2**	**Model 3**	**Model 4**
	**OR (95% CI)**	**AOR (95% CI)**	**AOR (95% CI)**	**AOR (95% CI)**	**AOR (95% CI)**
***Age***					
15-19	1.00 (Reference)	1.00 (Reference)	1.00 (Reference)	1.00 (Reference)	1.00 (Reference)
20-24	1.11(0.94-1.30)	1.36*(1.08-1.72)	1.60*(1.20-2.14)	1.60*(1.20-2.14)	1.60*(1.20-2.14)
25-29	1.53*(1.30-1.80)	1.84*(1.43-2.36)	2.34*(1.72-3.19)	2.36*(1.73-3.22)	2.34*(1.72-3.19)
30-34	1.71*(1.45-2.03)	2.27*(1.73-2.94)	3.06*(2.20-4.22)	3.06*(2.23-4.22)	3.06*(2.20-4.22)
35-39	2.21*(1.85-2.63)	2.69*(2.03-3.56)	4.01*(2.89-5.58)	4.01*(2.89-5.64)	4.01*(2.89-5.58)
40-44	2.48*(2.06-3.00)	3.63*(2.72-4.85)	5.75*(4.06-8.25)	5.75*(4.06-8.25)	5.75*(4.06-8.25)
45-49	3.13*(2.58-3.80)	4.44*(3.32-5.93)	7.69*(5.37-11.02)	7.69*(5.37-11.02)	7.69*(5.37-11.02)
***Region***					
North Eastern	1.00 (Reference)	1.00 (Reference)	1.00 (Reference)	1.00 (Reference)	1.00 (Reference)
Central	0.00*(0.00-0.01)	0.05*(0.02-0.09)	0.02*(0.00-0.32)	0.04*(0.00-3.42)	0.02*(0.00-0.33)
Coast	0.01*(0.00-0.01)	0.01*(0.00-0.01)	0.00*(0.00-0.03)	0.01*(0.00-0.30)	0.00*(0.00-0.03)
Eastern	0.00*(0.00-0.01)	0.07*(0.04-0.13)	0.04*(0.00-0.61)	0.04*(0.00-1.80)	0.04*(0.00-0.63)
Nairobi	0.02*(0.01-0.03)	0.03*(0.01-0.05)	0.01*(0.00-1.21)	0.02*(0.00-9.58)	0.01*(0.00-1.25)
Nyanza	0.01*(0.01-0.02)	0.06*(0.03-0.10)	0.02*(0.00-0.35)	0.03*(0.00-4.10)	0.02*(0.00-0.35)
Rift Valley	0.01*(0.01-0.02)	0.07*(0.04-0.12)	0.02*(0.00-0.30)	0.03*(0.00-2.69)	0.02*(0.00-0.31)
Western	0.00*(0.00-0.00)	0.00*(0.00-0.00)	0.00*(0.00-0.01)	0.00*(0.00-0.59)	0.00*(0.00-0.01)
***Type of place of residence***					
Urban	1.00 (Reference)	1.00 (Reference)	1.00 (Reference)	1.00 (Reference)	1.00 (Reference)
Rural	2.40*(2.14-2.68)	0.98(0.79-1.22)	0.90(0.68-1.19)	0.90(0.68-1.19)	0.90(0.68-1.19)
***Education***					
No formal	1.00 (Reference)	1.00 (Reference)	1.00 (Reference)	1.00 (Reference)	1.00 (Reference)
Primary	0.15*(0.13-0.18)	0.58*(0.46-0.73)	0.61*(0.45-0.80)	0.61*(0.46-0.81)	0.61*(0.45-0.80)
Secondary	0.09*(0.08-0.11)	0.44*(0.34-0.57)	0.30*(0.21-0.41)	0.30*(0.21-0.41)	0.30*(0.21-0.41)
Higher	0.06*(0.04-0.07)	0.32*(0.23-0.46)	0.20*(0.13-0.31)	0.20*(0.13-0.31)	0.20*(0.13-0.31)
***Marital status***					
Never Married	1.00 (Reference)	1.00 (Reference)	1.00 (Reference)	1.00 (Reference)	1.00 (Reference)
Married, living together	2.36*(2.10-2.65)	1.38*(1.14-1.65)	1.65*(1.31-2.05)	1.63*(1.31-2.05)	1.65*(1.31-2.05)
Separated	1.90*(1.59-2.27)	0.95(0.73-1.23)	1.30(0.96-1.75)	1.30(0.96-1.75)	1.30(0.96-1.75)
***Religion***					
Muslim	1.00 (Reference)	1.00 (Reference)	1.00 (Reference)	1.00 (Reference)	1.00 (Reference)
Roman Catholic	0.26*(0.22-1.00)	0.24*(0.18-0.32)	0.17*(0.12-0.25)	0.18*(0.12-0.25)	0.17*(0.12-0.25)
Other Christian	0.17*(0.15-0.19)	0.20*(0.15-0.27)	0.13*(0.09-0.18)	0.13*(0.09-0.19)	0.13*(0.09-0.18)
No religion	0.39*(0.28-0.56)	0.31*(0.19-0.49)	0.20*(0.11-0.36)	0.20*(0.11-0.37)	0.20*(0.11-0.36)
Other	0.06*(0.02-0.14)	0.04*(0.01-0.13)	0.03*(0.01-0.10)	0.03*(0.01-0.10)	0.03*(0.01-0.10)
***Socioeconomic status***					
Poorest	1.00 (Reference)	1.00 (Reference)	1.00 (Reference)	1.00 (Reference)	1.00 (Reference)
Poorer	0.42*(0.36-0.50)	1.08(0.88-1.34)	0.97(0.75-1.26)	0.98(0.75-1.27)	0.97(0.75-1.26)
Middle	0.37*(0.32-0.43)	0.88(0.71-1.08)	0.80(0.61-1.04)	0.81(0.62-1.05)	0.80(0.61-1.04)
Richer	0.31*(0.27-0.36)	0.78*(0.63-0.98)	0.71*(0.54-0.95)	0.72*(0.54-0.95)	0.71*(0.54-0.95)
Richest	0.17*(0.15-0.20)	0.66*(0.50-0.89)	0.64*(0.44-0.92)	0.64*(0.44-0.92)	0.64*(0.44-0.92)
***Media exposure***					
low	1.00 (Reference)	1.00 (Reference)	1.00 (Reference)	1.00 (Reference)	1.00 (Reference)
mid	0.35*(0.31-0.40)	0.67*(0.58-0.79)	0.89(0.73-1.07)	0.89(0.73-1.07)	0.89(0.73-1.07)
high	0.20*(0.17-0.22)	0.60*(0.49-0.73)	0.70*(0.55-0.90)	0.70*(0.55-0.90)	0.70*(0.55-0.90)
***Occupation***					
Not working	1.00 (Reference)	1.00 (Reference)	1.00 (Reference)	1.00 (Reference)	1.00 (Reference)
management	0.59*(0.51-0.68)	0.83(0.68-1.00)	0.93(0.74-1.17)	0.93(0.74-1.17)	0.93(0.74-1.17)
Other	0.92(0.83-1.02)	1.07(0.92-1.23)	1.07(0.90-1.28)	1.07(0.90-1.28)	1.07(0.90-1.28)
Random Effect					
Unstructured (*τ*_*u*_)			0.25(0.15-0.40)		0.25(0.15-0.40)
Structured (*τ*_*s*_)				0.06(0.04-0.10)	13910(1416-73690)
DIC		6889.79	5144.13	5142.92	5144.23
pD		31.84	68.54	68.2	68.59

*p < 0.05.

**Table 3:** Odds ratios, Adjusted Odds ratios and 95% Credible Intervals (CI) for the association between Continued FGM/C and the significant predictors

**Variable**		**Model 1**	**Model 2**	**Model 3**	**Model 4**
	**OR (95% CI)**	**AOR (95% CI)**	**AOR (95% CI)**	**AOR (95% CI)**	**AOR (95% CI)**
***Circumcised***					
No	1.00 (Reference)	1.00 (Reference)	1.00 (Reference)	1.00 (Reference)	1.00 (Reference)
Yes	29.67*(24.78-35.87)	10.5*(8.02-13.89)	10.5*(8.01-13.88)	10.2*(7.82-13.52)	10.53*(8.02-10.52)
***Age***					
15-19	1.00 (Reference)	1.00 (Reference)	1.00 (Reference)	1.00 (Reference)	1.00 (Reference)
20-24	0.95(0.79-1.15)	1.00(0.72-1.41)	1.00(0.72-1.41)	1.00(0.72-1.41)	1.00(0.72-1.00)
25-29	0.96(0.79-1.17)	0.69(0.48-1.01)	0.69(0.48-1.01)	0.70(0.48-1.01)	0.69(0.48-0.69)
30-34	0.78(0.63-0.96)	0.63*(0.42-0.95)	0.63*(0.42-0.95)	0.64*(0.42-0.96)	0.63*(0.42-0.63)
35-39	1.07(0.85-1.32)	0.66(0.43-1.00)	0.66(0.43-1.00)	0.66(0.43-1.00)	0.66(0.43-0.66)
40-44	0.82(0.63-1.05)	0.40*(0.25-0.62)	0.40*(0.25-0.63)	0.40*(0.25-0.63)	0.40*(0.25-0.40)
45-49	0.93(0.72-1.20)	0.44*(0.28-0.68)	0.44*(0.28-0.68)	0.44*(0.28-0.69)	0.44*(0.28-0.44)
***Region***					
North Eastern	1.00 (Reference)	1.00 (Reference)	1.00 (Reference)	1.00 (Reference)	1.00 (Reference)
Central	0.00*(0.00-0.01)	0.07*(0.02-0.22)	0.07*(0.02-0.22)	0.16*(0.03-0.95)	0.07*(0.02-0.07)
Coast	0.00*(0.00-0.01)	0.03*(0.01-0.09)	0.03*(0.01-0.09)	0.08*(0.02-0.38)	0.03*(0.01-0.03)
Eastern	0.02*(0.01-0.03)	0.10*(0.03-0.29)	0.10*(0.03-0.29)	0.20*(0.05-0.84)	0.10*(0.03-0.10)
Nairobi	0.00*(0.00-0.01)	0.10*(0.02-0.57)	0.10*(0.02-0.56)	0.16*(0.02-1.51)	0.10*(0.02-0.10)
Nyanza	0.02*(0.01-0.03)	0.19*(0.06-0.58)	0.19*(0.06-0.58)	0.81*(0.11-5.98)	0.19*(0.06-0.19)
Rift Valley	0.01*(0.00-0.01)	0.04*(0.01-0.10)	0.04*(0.01-0.10)	0.12*(0.02-0.67)	0.04*(0.01-0.04)
Western	0.00*(0.00-0.00)	0.05*(0.01-0.17)	0.05*(0.01-0.17)	0.22*(0.03-1.98)	0.05*(0.01-0.05)
***Type of place of residence***					
Urban	1.00 (Reference)	1.00 (Reference)	1.00 (Reference)	1.00 (Reference)	1.00 (Reference)
Rural	2.39*(2.14-2.69)	0.65*(0.44-0.94)	0.65*(0.44-0.94)	0.65*(0.45-0.95)	0.65*(0.44-0.65)
***Education***					
No formal	1.00 (Reference)	1.00 (Reference)	1.00 (Reference)	1.00 (Reference)	1.00 (Reference)
Primary	0.07*(0.06-0.08)	0.42*(0.31-0.57)	0.42*(0.31-0.57)	0.43*(0.32-0.58)	0.42*(0.31-0.42)
Secondary	0.05*(0.04-0.06)	0.34*(0.24-0.50)	0.34*(0.24-0.50)	0.35*(0.24-0.52)	0.34*(0.24-0.34)
Higher	0.02*(0.01-0.03)	0.18*(0.10-0.33)	0.18*(0.10-0.33)	0.19*(0.10-0.34)	0.18*(0.10-0.18)
***Marital status***					
Never Married	1.00 (Reference)	1.00 (Reference)	1.00 (Reference)	1.00 (Reference)	1.00 (Reference)
Married, living together	1.67*(1.45-1.93)	1.40*(1.04-1.89)	1.40*(1.04-1.89)	1.40*(1.04-1.89)	1.40*(1.04-1.40)
Separated	1.15(0.90-1.46)	1.50(0.99-2.25)	1.50(0.99-2.25)	1.50(0.99-2.25)	1.50(0.99-1.50)
***Religion***					
Muslim	1.00 (Reference)	1.00 (Reference)	1.00 (Reference)	1.00 (Reference)	1.00 (Reference)
Roman Catholic	0.09*(0.07-0.11)	0.64*(0.43-0.97)	0.64*(0.43-0.97)	0.65*(0.43-0.98)	0.64*(0.43-0.64)
Other Christian	0.06*(0.05-0.07)	0.49*(0.33-0.73)	0.49*(0.33-0.73)	0.49*(0.33-0.73)	0.49*(0.33-0.49)
No religion	0.31*(0.21-0.46)	1.95*(1.04-3.63)	1.95*(1.04-3.63)	1.99*(1.07-3.69)	1.95*(1.04-1.95)
Other	0.09(0.03-0.22)	1.37(0.30-4.81)	1.37(0.30-4.82)	1.39(0.30-4.86)	1.32(0.30-1.37)
***Socioeconomic status***					
Poorest	1.00 (Reference)	1.00 (Reference)	1.00 (Reference)	1.00 (Reference)	1.00 (Reference)
Poorer	0.25*(0.20-0.30)	0.92(0.67-1.27)	0.92(0.67-1.27)	0.93(0.68-1.27)	0.92(0.67-0.92)
Middle	0.22(0.18-0.27)	1.17(0.85-1.63)	1.17(0.85-1.63)	1.18(0.85-1.63)	1.17(0.85-1.17)
Richer	0.18(0.15-0.22)	0.87(0.61-1.25)	0.87(0.61-1.25)	0.87(0.61-1.25)	0.87(0.61-0.87)
Richest	0.13(0.11-0.15)	0.83(0.51-1.36)	0.83(0.51-1.36)	0.84(0.51-1.37)	0.83(0.51-0.83)
***Media Exposure***					
low	1.00 (Reference)	1.00 (Reference)	1.00 (Reference)	1.00 (Reference)	1.00 (Reference)
mid	0.12*(0.10-0.15)	0.84(0.66-1.07)	0.84(0.66-1.07)	0.85(0.67-1.08)	0.84*(0.66-0.84)
high	0.21*(0.18-0.24)	0.68*(0.50-0.93)	0.68*(0.50-0.93)	0.68*(0.50-0.93)	0.68*(0.50-0.68)
***Occupation***					
Not working	1.00 (Reference)	1.00 (Reference)	1.00 (Reference)	1.00 (Reference)	1.00 (Reference)
management	0.32*(0.30-0.35)	0.81(0.59-1.11)	0.81(0.59-1.11)	0.82(0.59-1.12)	0.81*(0.59-0.81)
Other	0.32*(0.26-0.39)	0.83(0.66-1.05)	0.83(0.66-1.05)	0.84(0.66-1.06)	0.83*(0.66-0.83)
Random Effect					
Unstructured (*t*_*u*_)			1.97(1.05-3.63)		1.97(1.05-3.63)
Structured (*t*_*s*_)				0.52(0.27-1.01)	13850(1399–73250)
DIC		3489.83	3363.04	3361.83	3363.36
pD		32.61	59.75	58.29	59.89

*p < 0.05.

The adjusted results suggest that all predictors considered, except the type of place of residence and the woman’s occupation, were significantly associated with FGM/C status. The results suggested a linear increase in FGM/C risk with age. We also found a linear decline in FGM/C risk with education, socioeconomic status and media exposure. The odds of women with primary education (AOR: 0.61, 95% CI: 0.46 -0.81) having undergone FGM/C were 39% lower than those for women with no formal education. The risk of having undergone the cut declines further among better educated women. Women with secondary education (AOR: 0.30, 95% CI: 0.21 -0.41) and those with tertiary education (AOR: 0.20, 95% CI: 0.13 -0.31) were at 70% and 80% lower risk, respectively, of having undergone the cut compared to women with no formal education.

We also noted that women with high (AOR: 0.70, 95% CI: 0.55 -0.90) and moderate (AOR: 0.89, 95% CI: 0.73 -1.07) media exposure were at 30% and 11% lower risk respectively, of having undergone the procedure.

In terms of the response to the question concerning the continuation of the practice, all predictors except marital status, socioeconomic status and occupation were found to be significant. Women in the North Eastern province expressed greater support for the continuation of FGM/C compared to women from other provinces in Kenya. The results also suggest that women who had already undergone FGM/C were more likely to support the continuation of the practice compared to those women who had not (AOR: 10.96, 95% CI: 8.34-14.50). We found a linear relationship between age and support for the continuation of FGM/C. The results however suggest that it is only women aged 40–44 years (AOR: 0.45, 95% CI: 0.30-0.67) and 45–49 years (AOR: 0.48, 95% CI: 0.32-0.71) who demonstrated significantly lower support for the continuation of FGM/C, compared to women aged 15–19 years. Support for the continuation of FGM/C was also much lower among women in urban areas (AOR: 0.66, 95% CI: 0.49-0.88) compared to women in rural settings.

We also found significant linear decline in support for the continuation of the practice with education and media exposure.

### Spatial clustering of FGM/C

#### Spatial clustering of women circumcised

Table 4 presents the results of the spatial scan statistical analysis. A spatial scan statistic was applied to the aggregated county level data and identified six main high-risk FGM/C clusters. The first cluster (RR = 3.93, p < 0.001) identified consisted of 779 (257.64 expected) cases in six counties. This cluster consisted of Mandera, Wajir, Marsabit, Isiolo, Garissa and Samburu counties. The second significant cluster (RR = 2.97, p < 0.001) identified consisted of 474 (182.09 expected) cases in four counties namely, Bomet, Nyamira, Kisii and Narok and the third cluster with 109 (61.01 expected) cases, with a relative risk of 1.82 (p < 0.001), consisted of Kitui county. These clusters are in regions inhabited by the Somali, Kisii, Maasai, Kuria and Meru ethnic communities.

**Table 4:** Statistically significant high-risk clusters

***Outcome***	***Type***		**Observed (Expected) cases**	**Relative risk (p-value)**	**Counties**
*Woman circumcised*	*High risk cluster*	Most likely cluster	779(257.64)	3.92(<0.001)	Mandera, Wajir, Marsabit, Isiolo, Garissa, Samburu
		Secondary cluster	474(182.09)	2.97(<0.001)	Bomet, Nyamira, Kisii, Narok
		Third most likely cluster	109(61.01)	1.82(<0.001)	Kitui
		Fourth most likely cluster	52(25.29)	2.08(<0.001)	Baringo
		Fifth most likely cluster	29(12.96)	2.25(<0.001)	WestPokot
		Sixth most likely cluster	205(154.90)	1.35(0.02)	Meru, Tharaka-Nithi, Embu
	*Low risk cluster*	Most likely cluster	45(558.91)	0.064(<0.001)	Busia, Siaya, Kakamega, Bungoma, Vihiga, Kisumu, Nandi, HomaBay
		Secondary cluster	488(844.68)	0.48(<0.001)	Taita Taveta, Kwale, Kilifi, Mombasa, Makueni, Kitui, Kajiado, Tana River, Machakos, Nairobi, Lamu, Kiambu
*FGM/C should continue*	*High risk cluster*	Most likely cluster	702(54.84)	12.80(<0.001)	Mandera, Wajir, Marsabit, Isiolo, Garissa
		Secondary cluster	153(59.37)	2.58(<0.001)	Kisii, Nyamira
	*Low risk cluster*	Most likely cluster	18(166.31)	0.08(<0.001)	Kakamega, Vihiga, Bungoma, Nandi, Busia, Kisumu, Uasin Gishu, Siaya, Trans Nzoia, Kericho, Elgeyo Marakwet, HomaBay
		Secondary cluster	66(218.94)	0.20(<0.001)	TaitaTaveta, Kwale, Kilifi, Mombasa, Makueni, Kitui, Kajiado, TanaRiver, Machakos, Nairobi, Lamu, Kiambu, Embu, Muranga, Kirinyaga, Tharaka-Nithi, Nyeri

Two low-risk clusters were identified. The main low-risk cluster, with a relative risk of 0.06 (p < 0.001), consisted of counties in Western and Nyanza provinces whereas the secondary cluster identified, with a relative risk of 0.48 (p < 0.001), consisted of counties in the Coast and Eastern provinces.

#### Spatial clustering of support for the continuation of FGM

With regards to support for the continuation of FGM/C, two significant clusters were identified. This first cluster (RR = 12.80, p < 0.001) identified consisted of 702 (54.84 expected) cases in five counties. This cluster consisted of Mandera, Wajir, Marsabit, Isiolo, and Garissa counties. The next significant cluster (RR = 2.58, p < 0.001) identified consisted of 153 (59.37 expected) cases in two counties namely Kisii and Nyamira. These clusters are in regions inhabited by the Somali, Kisii, and Meru ethnic communities.

### Spatial mapping

**Figure 1:**
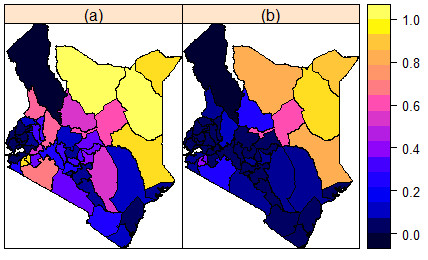
**Observed (Crude) county level prevalence maps. (a)** Prevalence of FGM/C. **(b)** Proportion of women in support of FGM/C continuation.

Figure 1 presents relative risk maps of FGM/C and of prevalence of support for the continuation of FGM. It is apparent from these maps that FGM/C ‘hotspots’ are in the North Eastern and South Western regions of the country.

**Figure 2:**
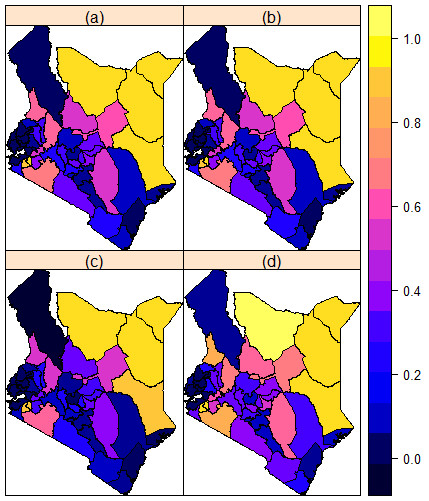
**Prevalence of FGM/C predicted from the parsimonious spatial model. (a)** Mean posterior prevalence rates. **(b)** Median posterior prevalence rates. **(c)** 2.5% posterior prevalence rates. **(d)** 97.5% posterior prevalence rates.

Figure 2 presents smoothed FGM/C risk maps for Kenya that arose from fitting the hierarchical spatial model with a spatially structured random effect to the data. It is apparent yet again that the North Eastern and South Western regions of the country bear the greatest FGM/C burden.

**Figure 3:**
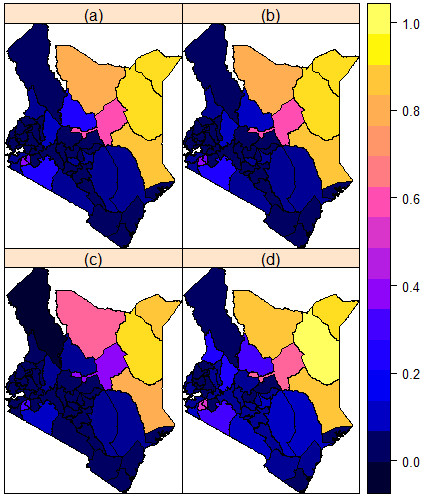
**Prevalence of support for FGM/C continuation predicted from the parsimonious spatial model. (a)** Mean posterior prevalence rates. **(b)** Median posterior prevalence rates. **(c)** 2.5% posterior prevalence rates. **(d)** 97.5% posterior prevalence rates.

In Figure 3 we present a smoothed map of the prevalence of support for FGM/C in Kenya. This map also supports the results of the spatial cluster analysis.

## Discussion

This study used a Bayesian hierarchical spatial modelling approach to investigate the spatial variation in the risk and intention to continue the practice of FGM/C in Kenya. As opposed to a more standard Markov Chain Monte Carlo approach, we employed an Integrated Nested laplace algorithm within the R library INLA to fit Besag, York and Mollie [45] like models. The Kulldorff spatial scan statistic was used for cluster detection and to test for local clusters of FGM/C. The results of the spatial scan statistical analysis supported and confirmed the results of the findings of the Bayesian hierarchical analysis.

The study has demonstrated both geographical heterogeneity in the practice, and support for the continued practice of FGM/C in Kenya. The counties located in the North Eastern and South Western parts of the country are identified as FGM ‘hotspots’ and are areas in need of urgent attention. These findings reveal the enormity of the task faced by the Government of Kenya, in support of initiatives by World Health Organization (WHO), to outlaw the practice in the country [1].

This study finds a linear negative relationship between a woman’s level of education and the view that FGM/C should continue. Similar results have been reported elsewhere [21,24,32] and are consistent with modernization theory that argues that education transmits Western ideals, in particular individual rights and the importance of health [21], thereby empowering women to make informed decisions concerning their well-being. The results are also consistent with the content aspect of conventional theory where educated women are considered more likely to learn about the negative consequences of female genital cutting and are therefore less likely to support its continuation [24,27]. As a consequence educated women are better able to make decisions and to seek alternative opportunities for their daughters.

As indicated in other studies in the literature, the decision to have one’s child circumcised is never the prerogative of the woman alone and is in most settings driven by societal, household, husband and family considerations [24,30,46]. Initial results from the study suggest that women who are better educated possibly have a greater say in the health issues that affect their families, and are less likely to support the continuation of the practice, compared to those whose husband or others have made the decisions regarding the family’s health. A limitation of this study stems from the fact that the DHS data contained limited information about the role of household members in decisions concerning female genital mutilation. This study therefore, does not fully account for the decision making dynamics within households.

The results of the study indicate high prevalence and clusters of the practice in counties within the North Eastern province of Kenya, bordering Somalia and Ethiopia, parts of South Nyanza, near Lake Victoria, and in parts of the Eastern province. The study also adds to the general body of knowledge on the subject of women’s support for FGM/C by documenting and mapping prevalence and identifying high-risk clusters. The fact that there were no high-risk clusters in support of the continuation of FGM/C in those communities with a current high prevalence of FGM/C, needs mentioning. The study did not find significant clusters of support for the continuation of FGM/C in counties where the Meru (Meru North, Tharaka), Kikuyu (Muranga, Thika, Kirinyaga, Kiambu County), Kamba (Mwingi county) and Taita (Taita/Taveta county) ethnic groups live. This, as noted by others [24], suggests a possible decline, or eradication of the practice amongst these communities. There is a need to carry out a spatio-temporal analysis to confirm this assertion. The relationship between ethnicity and FGM/C is however quite complex and the break from, or maintenance of, traditional behaviours requires further investigation [24,47].

The cause of FGM/C has been explained as a mix of cultural, religious and social factors [26,29,31]. It is considered an obligatory social and traditional norm aimed at maintaining virginity and sexual chastity, and the reduction and control of female sexuality [3,25,28]. Controversial and safer clinical approaches have been suggested and are practiced in many communities worldwide in the hope that these positive approaches rather than censorious ones may lead to a more rapid decline in FGM/C prevalence [48]. Current strategies geared towards reduction in FGM/C prevalence are shifting from the more clinical and health risk approaches to models that seek to address the phenomena within its social context. Communities in Kenya, and other countries across the globe where FGM/C is rampant, are structured along patriarchal systems that control both sexuality and fertility [49]. The social pressure to conform has been identified as a strong motivator in perpetuating the practice [23]. It is often considered a necessary part of raising a girl properly, and a way of preparing her for adulthood and marriage, influenced by beliefs linking the procedures to premarital virginity and marital fidelity. Although there exists no religious scripts that prescribe the practice, practitioners often believe the practice has religious support [50,51]. The results of this study indicated clustering of FGM/C in the North Eastern province of Kenya where Islam is a dominant religion. As with the other FGM/C clusters identified, the practice was prevalent is regions with poor access to basic education, extreme poverty, and low media exposure [52,53]. There is evidence based on our findings that FGM/C continues unabated in sparsely populated and remote regions that are quite isolated from the rest of the country and given low priority in national development efforts.

In Kenya, the practice was nearly universal among the Kisii and Maasai and very common among Kalenjin, Taita/Taveta, Embu/Mere, and to a lesser extent among the Kikuyu, Kamba, and Mjikenda/Swahili [54]. Our results concur with these finding but also indicate a possible high prevalence of the practice amongst the Somali, Rendile, Borana and Oromo community in North Eastern Province, Kenya. FGM/C is however not practiced among some ethnic groups in the country, notably the Luo, Luhya, Teso and Turkana.

Although the political and legal environment in Kenya has been hostile towards the practice, the results of this study indicate that it will take a great effort to see a substantial and sustainable change in the prevalence of the practice in certain regions and sectors. Despite the defeat in parliament of a motion to outlaw some forms of FGM/C in 1996, the Kenyan government formally outlawed the practice by passing the 2001 Children’s Bill [55].

Numerous FGM/C abandonment interventions have been proposed in the literature. Each intervention was designed to address a given context and target factors (tradition, religion and reduction in female sexual desire) assumed to support the continuation of FGM. These included human rights based approaches, public declarations, legislative mechanisms, alternative rites of passage, national and regional workshops, training and conversion of circumcisers, training of trainer workshops and comprehensive social development processes [56-59]. The rates of success of these interventions have varied significantly, based on context and whether they are perceived by communities to respect socio-cultural values and settings. Efforts to eradicate the practice in Kenya must target the high-risk FGM/C clusters identified in this study, but also need to effectively involve community, religious and political leaders in order to achieve meaningful change.

## Conclusions

The fight against FGM/C in Kenya is not over. There are still deep cultural and religious beliefs alongside illiteracy that have made it difficult to completely eradicate the practice. Interventions by government and other stakeholders must address these challenges and target the clusters identified in this study in order to achieve the goals set out by the Committee on the Elimination of all Forms of Discrimination against Women. The education of the girl child, along with empowerment to make informed decisions on personal health, must be given priority in the regions identified and in the nation as a whole.

### Consent

Informed consent of the respondent was obtained for the survey at the start of the individual interview. A standard consent form approved by the Scientific and Ethical Review Committee of the Kenya Medical Research Institute (KEMRI) and by the Ethics Committee of the Opinion Research Corporation Macro International Incorporated (ORC Macro Inc.), Calverton, USA was read to the respondent in their native language. Once the respondent agreed to participate in the survey, the interviewer confirmed this consent and signed the consent form indicating that the respondent had agreed to participate in the survey.

## Appendix

We assume that *Y*_*i*_ is a dichotomous variable taking value 1, if the *i-*th woman has undergone FGM/C and 0, otherwise, *i*, *i* = 1, …, 8444. We assumed that variable is Bernoulli with an unknown probability *p* that the woman has undergone the cut. That is, *Y*_*i*_ ∼ *Bern*(*p*_*i*_) and so we model the risk of FGM/C using a Hierarchical spatial logistic regression model that accounts for excess heterogeneity and spatial similarity between counties. The effects of covariates of different types were modelled as follows:

$$\mathrm{logit}\left(p_{i}\right)=\beta_{0}+X_{i}^{\prime }\beta +f_{s}\left(s_{i}\right)+f_{u}\left(s_{i}\right),$$

where ***β*** = (*β*_*1*_, …, *β*_*p*_)′ is the (*p* × 1) vector of parameter estimates, **X**_*i*_ corresponding linear effects of covariates, *f*_*u*_(*s*_*i*_) is a spatial unstructured component, which is independent and identically distributed with zero mean and unknown precision, *τ*_*u*_, and *f*_*s*_(*s*_*i*_) is spatially structured component which is assumed to vary smoothly from region to region. To account for such smoothness *f*_*s*_(*s*_*i*_) is modelled as an intrinsic Gaussian Markov random field with unknown precision, *τ*_*s*_, [60]. This is the usual conditionally autoregressive prior [35]. The latent Gaussian field for this model is *ξ* = {*β*_0_, {*β*_*j*_}, {*f*_*s*_(.)}, {*f*_*u*_(.)}, {*p*_*i*_}} with hyperparameter vector *ϑ* = {*τ*_*β*_, *τ*_*u*_, *τ*_*s*_}. Vague independent Gamma priors are assigned to each of the elements in *ϑ*.

A similar model was also applied to the case where the response was the dichotomous variable taking a value 1 if the woman felt that FGM/C *Should FGM/C be continued* and *0* otherwise.

## Competing interests

The author declares that he has no competing interests.

## Pre-publication history

The pre-publication history for this paper can be accessed here:

http://www.biomedcentral.com/1471-2458/14/276/prepub

## References

[B1] Bjälkander O Bangura L Leigh B Berggren V Bergström S Almroth L Health complications of female genital mutilation in Sierra Leone International Journal of Women’s Health 2012 4 321 331 10.2147/IJWH.S32670 PMC3410700 22870046

[B2] World Health Organization Female genital mutilation, Media Centre; 2000. Fact sheet N°241 2000 Available from: http://collections.infocollections.org/ukedu/en/d/Js0519e/

[B3] Rymer J Female genital mutilation Curr Obstet Gynecol 2003 13 3 185 190 10.1016/S0957-5847(03)00004-0

[B4] Toubia N Female circumcision as a public health issue N Engl J Med 1994 331 11 712 716 10.1056/NEJM199409153311106 8058079

[B5] Almroth L Bedri H el Musharaf S Satti A Idris T Hashim MSK Suliman GI Bergström S Urogenital complications among girls with genital mutilation: a hospital-based study in Khartoum Afr J Reprod Health 2005 9 2 118 124 10.2307/3583468 16485592

[B6] Behrendt A Moritz S Posttraumatic stress disorder and memory problems after female genital mutilation Am J Psychiatry 2005 162 5 1000 1002 10.1176/appi.ajp.162.5.1000 15863806

[B7] Dare F Oboro V Fadiora S Orji E Sule-Odu A Olabode T Female genital mutilation: an analysis of 522 cases in South-Western Nigeria J Obstet Gynecol 2004 24 3 281 283 10.1080/01443610410001660850 15203627

[B8] Kaplan A Hechavarría S Martín M Bonhoure I Health consequences of female genital mutilation/cutting in the Gambia, evidence into action Reprod Health 2011 8 26 1 6 21967670 10.1186/1742-4755-8-26 PMC3195700

[B9] Morison L Scherf C Ekpo G Paine K West B Coleman R Walraven G The long‒term reproductive health consequences of female genital cutting in rural Gambia: a community‒based survey Trop Med Int Health 2001 6 8 643 653 10.1046/j.1365-3156.2001.00749.x 11555430

[B10] Dirie M Lindmark G The risk of medical complications after female circumcision East Afr Med J 1992 69 9 479 482 1286628

[B11] Abd El-Naser T Farouk A El-Nashar AE-R Mostafa T Sexual side effects of female genital mutilation/cutting May Be type dependent: a hospital-based study J Obstet Gynecol 2011 1 2 65 74

[B12] Alsibiani SA Rouzi AA Sexual function in women with female genital mutilation Fertil Steril 2010 93 3 722 724 10.1016/j.fertnstert.2008.10.035 19028385

[B13] Banks E Meirik O Farley T Akande O Bathija H Ali M Female genital mutilation and obstetric outcome: WHO collaborative prospective study in six African countries Lancet 2006 367 9525 1835 1841 16753486 10.1016/S0140-6736(06)68805-3

[B14] Dorkenoo E Combating female genital mutilation: an agenda for the next decade World Health Stat Q 1996 49 2 142 9050193

[B15] Eke N Nkanginieme KEO Female Genital Mutilation: A Global bug that should not cross the millennium bridge World J Surg 1999 23 10 1082 1087 10.1007/s002689900627 10512951

[B16] Kenya National Bureau of Statistics and ICF Macro Kenya Demographic and Health Survey 2003 2003 Calverton, Maryland: KNBS and ICF Macro

[B17] Kenya National Bureau of Statistics and ICF Macro Kenya Demographic and Health Survey 2008–09 2010 Calverton, Maryland: KNBS and ICF Macro

[B18] Benwell GL McLennan BR Visualising Spatial Pattern and Correlation: Observations from the Global Atlas on Violence and Health SIRC 99 – The 11-th Annual Colloquium of the Spatial Information: 1999 1999 Dunedin, New Zealand: University of Otago

[B19] Kandala NB Nwakeze N Kandala SNII Spatial distribution of female genital mutilation in nigeria Am J Trop Med Hyg 2009 81 5 784 792 10.4269/ajtmh.2009.09-0129 19861612

[B20] Kouba LJ Muasher J Female circumcision in Africa: an overview Afr Stud Rev 1985 28 1 95 110 10.2307/524569

[B21] Boyle EH McMorris BJ Gomez M Local conformity to international norms Int Sociol 2002 17 1 5 33 10.1177/0268580902017001001

[B22] Caldwell JC Orubuloye I Caldwell P Male and female circumcision in Africa from a regional to a specific Nigerian examination Soc Sci Med 1997 44 8 1181 1193 10.1016/S0277-9536(96)00253-5 9131742

[B23] MRS-EA Fact Sheet. Female genital mutilation MedicalKenya 2011 http://medicalkenya.co.ke/2011/02/fact-sheet-female-genital-mutilation/

[B24] Hayford SR Conformity and change: community effects on female genital cutting in Kenya J Health Soc Behav 2005 46 2 121 140 10.1177/002214650504600201 16028453

[B25] Jones SD Ehiri J Anyanwu E Female genital mutilation in developing countries: an agenda for public health response Eur J Obstet Gynecol Reprod Biol 2004 116 2 144 151 10.1016/j.ejogrb.2004.06.013 15358454

[B26] Klouman E Manongi R Klepp KI Self-reported and observed female genital cutting in rural Tanzania: associated demographic factors, HIV and sexually transmitted infections Trop Med Int Health 2005 10 1 105 115 10.1111/j.1365-3156.2004.01350.x 15655020

[B27] Mackie G Stokes C Castle S Heise L Raikes A Watts C Zwi A Makinwa P Jensen A Bankston C Ending footbinding and infibulation: a convention account American Sociological Review 1996 61 6 999 1017 10.2307/2096305

[B28] Mitike G Deressa W Prevalence and associated factors of female genital mutilation among Somali refugees in eastern Ethiopia: a cross-sectional study BMC Public Health 2009 9 1 264 10.1186/1471-2458-9-264 19635149 PMC2724517

[B29] Satti A Elmusharaf S Bedri H Idris T Hashim MSK Suliman G Almroth L Prevalence and determinants of the practice of genital mutilation of girls in Khartoum, Sudan Ann Trop Paediatr 2006 26 4 303 310 10.1179/146532806X152827 17132295

[B30] Shell-Duncan B Hernlund Y Women without choices: the debate over medicalization of female genital cutting and its impact on a northern Kenyan community Female "circumcision" in Africa: Culture, Controversy, and Change 2000 Lynne Rienner Publishers 109 128

[B31] Snow R Slanger T Okonofua FE Oronsaye F Wacker J Female genital cutting in southern urban and peri-urban Nigeria: self-reported validity, social determinants and secular decline Trop Med Int Health 2002 7 1 91 100 10.1046/j.1365-3156.2002.00829.x 11851959

[B32] Yount KM Like mother, like daughter? Female genital cutting in Minia, Egypt J Health Soc Behav 2002 43 3 336 358 10.2307/3090208 12467257

[B33] Dunteman GH Principal Component Analysis 1989 Newbury Park: SAGE publication

[B34] StataCorp L Stata Survey Data Reference Manual: Release 11 2009 College Station: Stata Press

[B35] Besag J York J Mollie A Bayesian image restoration with two applications in spatial statistics (with discussion) Ann Inst Stat Math 1991 43 1 59 10.1007/BF00116466

[B36] Best N Richardson S Thomson A A comparison of Bayesian spatial models for disease mapping Stat Methods Med Res 2005 14 1 35 59 10.1191/0962280205sm388oa 15690999

[B37] MacNab YC Hierarchical Bayesian spatial modelling of small‒area rates of non‒rare disease Stat Med 2003 22 10 1761 1773 10.1002/sim.1463 12720309

[B38] Mollie A Richardson S Empirical Bayes estimates of cancer mortality rates using spatial models Stat Med 1991 10 1 95 112 10.1002/sim.4780100114 2006359

[B39] Martino S Rue H R Package: INLA 2009 Norway: Department of Mathematical Sciences, NTNU Available at http://www.r-inla.org

[B40] Rue H Martino S Chopin N Approximate Bayesian inference for latent Gaussian models by using integrated nested Laplace approximations J Royal Stat Soc B 2009 71 2 319 392 10.1111/j.1467-9868.2008.00700.x

[B41] Rue HA Martino S Approximate Bayesian inference for hierarchical Gaussian Markov random field models J Stat Plann Inference 2007 137 10 3177 3192 10.1016/j.jspi.2006.07.016

[B42] Spiegelhalter DJ Best NG Carlin BP van der Linde A Bayesian measures of model complexity and fit J Royal Stat Soc B 2002 64 4 583 639 10.1111/1467-9868.00353

[B43] Kulldorff M SaTScan-Software for the spatial, temporal, and space-time scan statistics 2010 Boston: Harvard Medical School and Harvard Pilgrim Health Care

[B44] Anselin L Syabri I Kho Y GD An Introduction to Spatial Data Analysis Geogr Anal 2006 38 1 5 22 10.1111/j.0016-7363.2005.00671.x

[B45] Besag J York J Mollié A Bayesian image restoration, with two applications in spatial statistics Ann Inst Stat Math 1991 43 1 1 20 10.1007/BF00116466

[B46] Berg RC Denison E A tradition in transition: factors perpetuating and hindering the continuance of female genital mutilation/cutting (FGM/C) summarized in a systematic review Health care for women international 2013 34 10 837 859 10.1080/07399332.2012.721417 23489149 PMC3783896

[B47] Lockwood M Structure and behavior in the social demography of Africa Popul Dev Rev 1995 1 32

[B48] Valderrama J Female genital mutilation: why are we so radical? Lancet 2002 359 9305 529 530 11853834 10.1016/S0140-6736(02)07655-9

[B49] Toubia NF Sharief E Female genital mutilation: have we made progress? Int J Gynecol Obstet 2003 82 3 251 261 10.1016/S0020-7292(03)00229-7 14499972

[B50] World Health Organization Islamic ruling on male and female circumcision. The Right Path to Health, Health Education through Religion 1996 [Available from: http://applications.emro.who.int/dsaf/dsa54.pdf]

[B51] Masho SW Matthews L Factors determining whether Ethiopian women support continuation of female genital mutilation Int J Gynecol & Obstetrics 2009 107 3 232 235 10.1016/j.ijgo.2009.07.022 19716126

[B52] Achoka J Odebero S Maiyo J Ndiku J Access to basic education in Kenya: Inherent concerns Educ Res Rev 2007 2 10 275 284

[B53] Luseno WK McPeak JG Barrett CB Little PD Gebru G Assessing the value of climate forecast information for pastoralists: evidence from southern Ethiopia and northern Kenya World Dev 2003 31 9 1477 1494 10.1016/S0305-750X(03)00113-X

[B54] Lokurosia JC An assessment of the impact of health compaigns against female genital mutilation in west pokot district 2011 Kenya: Kenyatta University

[B55] Government of Kenya Children’s Bill 2001 Kenya Gazette Supplement No. 18 (Bill No. 4) Available from: http://www1.chr.up.ac.za/undp/domestic/docs/legislation_03.pdf

[B56] Berg RC Denison E Effectiveness of interventions designed to prevent female genital mutilation/cutting: A systematic review Stud Fam Plann 2012 43 2 135 146 10.1111/j.1728-4465.2012.00311.x 23175952

[B57] Brown K Beecham D Barrett H he Applicability of Behaviour Change in Intervention Programmes Targeted at Ending Female Genital Mutilation in the EU: Integrating Social Cognitive and Community Level Approaches Female Genital Mutilation, Cutting, or Circumcision 2013 New York, USA: Hindawi Publishing Corporation Available from: http://www.hindawi.com/journals/ogi/2013/324362/10.1155/2013/324362 PMC3745976 23983698

[B58] Feldman-Jacobs C Ryniak S Wilcher R Shears K Ellsberg M Abandoning female genital mutilation/cutting: an in-depth look at promising practices 2006 Washington, D.C: Population Reference Bureau

[B59] Oloo H Wanjiru M Newell-Jones K Female genital mutilation practices in Kenya: the role of alternative rites of passage: a case study of Kisii and Kuria districts 2011 London, United Kingdom: Feed the Minds

[B60] Rue H Held L Gaussian Markov Random Fields: Theory and Applications 2005 Boca Raton-London-New York-Singapore: Chapman & Hall/CRC

